# Severe hyperhomocysteinemia due to cystathionine β-synthase deficiency, and Factor V Leiden mutation in a patient with recurrent venous thrombosis

**DOI:** 10.1186/s12959-014-0030-0

**Published:** 2014-12-16

**Authors:** Zuhier Awan, Sumayah Aljenedil, David S Rosenblatt, Jean Cusson, Brian M Gilfix, Jacques Genest

**Affiliations:** King Abdulaziz University, Jeddah, Saudi Arabia; The McGill University Health Centre, Montreal, Canada; Department of Human Genetics, McGill University, Montreal, Canada; Department of Medicine, Université de Sherbrooke, Sherbrooke, Canada; Royal Victoria Hospital, 687 Pine Avenue West, Rm M4.72, Montreal, QC H3A 1A1 Canada

## Abstract

Homocysteine is an amino acid that is toxic to vascular endothelial cells, and plasma elevations have been associated with venous thromboembolism. Severe hyperhomocysteinemia (>100 μmol/L) may result from mutations in the genes coding for enzymes in the trans-sulfuration or the folate/vitamin B_12_-dependent re-methylation pathways. Here, we report the case of a young woman with severe, recurrent thrombo-embolic events associated with severe hyperhomocysteinemia (111 μmol/L). We identified a homozygous mutation in the cystathionine β -synthase gene (p.I278T) and the presence of the Factor V Leiden mutation. Family study shows segregation of elevated homocysteine in heterozygous relatives for the mutation in the cystathionine β -synthase gene. Management consisted of anticoagulation with warfarin and supplementation with folate, vitamin B_6_ (pyridoxine) and vitamin B_12_. After twelve years of follow-up, plasma homocysteine levels remain in the moderate range (~20 μmol/L, reference range 8-12 μmol/L) and no further thromboembolic events were identified.

## Introduction

Elevated free and protein-bond plasma homocysteine (tHcy) has been associated with venous thrombosis, pulmonary embolism and premature mortality. Methionine is a major donor of a methyl group required for a large variety of biological molecules and the resultant molecule, homocysteine, is toxic to vascular endothelial tissues. Homocysteine can be metabolized through the trans-sulfuration pathway. The enzyme cystathionine β-synthase (CBS) (EC 4.2.1.22) catalyses the condensation of homocysteine and serine to yield cystathionine, a reaction requiring pyridoxine (vitamin B_6_) as a cofactor. Cystathionine γ-lyase (EC 4.2.1.22) then converts cystathionine to cysteine and α-ketobutyrate. Alternatively, homocysteine can be recycled into methionine through the folate pathway. This process uses N_5_-methyltetrahydrofolate, S-Adenosylmethionine and methylcobalamin, with methylenetetrahydrofolate reductase (MTHFR) (EC 1.5.7.1) as the rate-limiting step [1]. Median plasma levels of tHcy levels in Western populations are 10 to 12 μmol/L with women having 10-15% lower homocysteine levels during their reproductive years.

## Case presentation

Here, we present a 30-year follow up on a woman with recurrent venous thromboembolism associated with both severe hyperhomocysteinemia, and the Factor V Leiden (FVL) mutation.

At the age of 21, a woman was referred to our institution with severe headache, nausea and vomiting. A CT scan of the head and lumbar puncture were not diagnostic. A diagnosis of a viral infection was made. While traveling abroad, her symptoms reappeared and were associated with decreased level of consciousness. On admission, a CT scan showed a hypo-dense lesion in the left thalamus, with no hydrocephalus or mass lesion. All cultures were negative and a presumptive diagnosis of viral infection was again made. The patient was started on acyclovir, a cephalosporin, and dexamethasone. The symptoms were alleviated and the patient was discharged.

After a few weeks, the patient suffered a relapse of her symptoms and loss of consciousness. On examination she was afebrile, had a blood pressure of 140/75, and the Glasgow coma scale score was 12 (moderate impairment; control is 15). She was noted to have roving eye movements, and disc margins were blurred on the nasal fields. Cranial nerves were intact. Hand grasp was weaker on the right side than on the left; the right upper limb was hyperreflexic. An angiogram showed occlusion of the intra-cerebral veins supplying the head of caudate. This was thought to be due to a thrombotic event. She was continued on steroid treatment and was discharged after her symptoms were stabilized. She was not put on systemic anticoagulation at that time.

**Figure 1:**
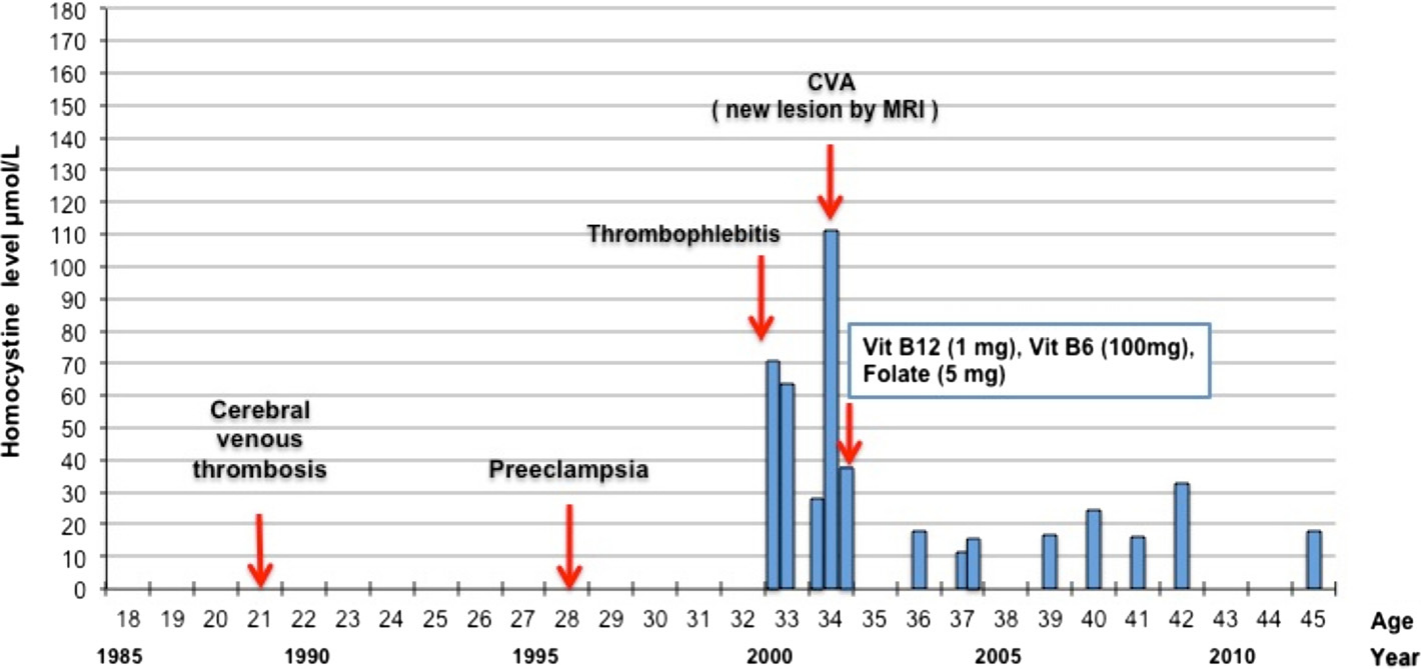
**Clinical course of index proband over 30 years.** The initial presentation of cerebral thrombosis, thrombophlebitis and cerebrovascular accident (CVA) led to further investigations. The patient was found to have very elevated total homocysteine (tHcy) levels and Factor V Leiden. Treatment with warfarin and folate, vitamin B_12_ and vitamin B_6_ resulted in a dramatic reduction of tHcy. Over a 12-year follow-up period, no further thrombo-embolic phenomena were observed.

At the age of 28, she had pre-eclampsia. She was treated with bisoprolol for post-partum hypertension. She developed thrombophlebitis at the age of 34 (Figure 1). At follow up, an MRI showed old hemorrhagic foci in the anterior limb of the left internal capsule, both thalami, and the left posterior superior frontal lobe.

Laboratory results showed levels of protein C and S within the reference ranges, and negative phospholipid antibodies. She was found to be heterozygous for the FVL mutation. Her tHcy level preceding her thrombophlebitis event was 54 μmol/L, and increased to 111 μmol/L within one year (reference range 4.6-15.5 μmol/L) (Figure 1 and Table 1). Plasma levels of vitamin B_12_, Folate and red blood cell folate were within reference ranges. Daily one time oral supplements of Vitamins B_12_ (1000 μg), vitamin B6 (100 mg), and folate (5 mg) were started, and later betaine (750 mg/kg) was added; her tHcy levels decreased to approximately 15-20 μmol/L.

**Table 1:** **Demographic characteristics and investigation results for the proband and her family**

**ID**	**Gender**	**Age**	**Total cholesterol**	**LDL**	**tHcy**	**CBS**	**MTHFR**	**FVL**
102	F	89	5.85	3.86	9.1	+ / -	+ / -	- / -
201	M	66	3.12	2.07	18.1	+ / -	- / -	- / -
202	F	67	6.11	4.04	19.3	+ / -	+ / -	+ / -
301 (Proband)	F	41	4.77	3.07	111	+ / +	- / -	+ / -
303	M	43	6.02	4.26	18.4	-	+ / -	- / -
401	M	13	3.14	1.75	11.4	-	+ / -	+ / -
412	F	12	4.72	3.31	8.4	-	- / -	- / -
410	F	6	5.16	3.57	8.1	-	- / -	- / -

**Figure 2:**
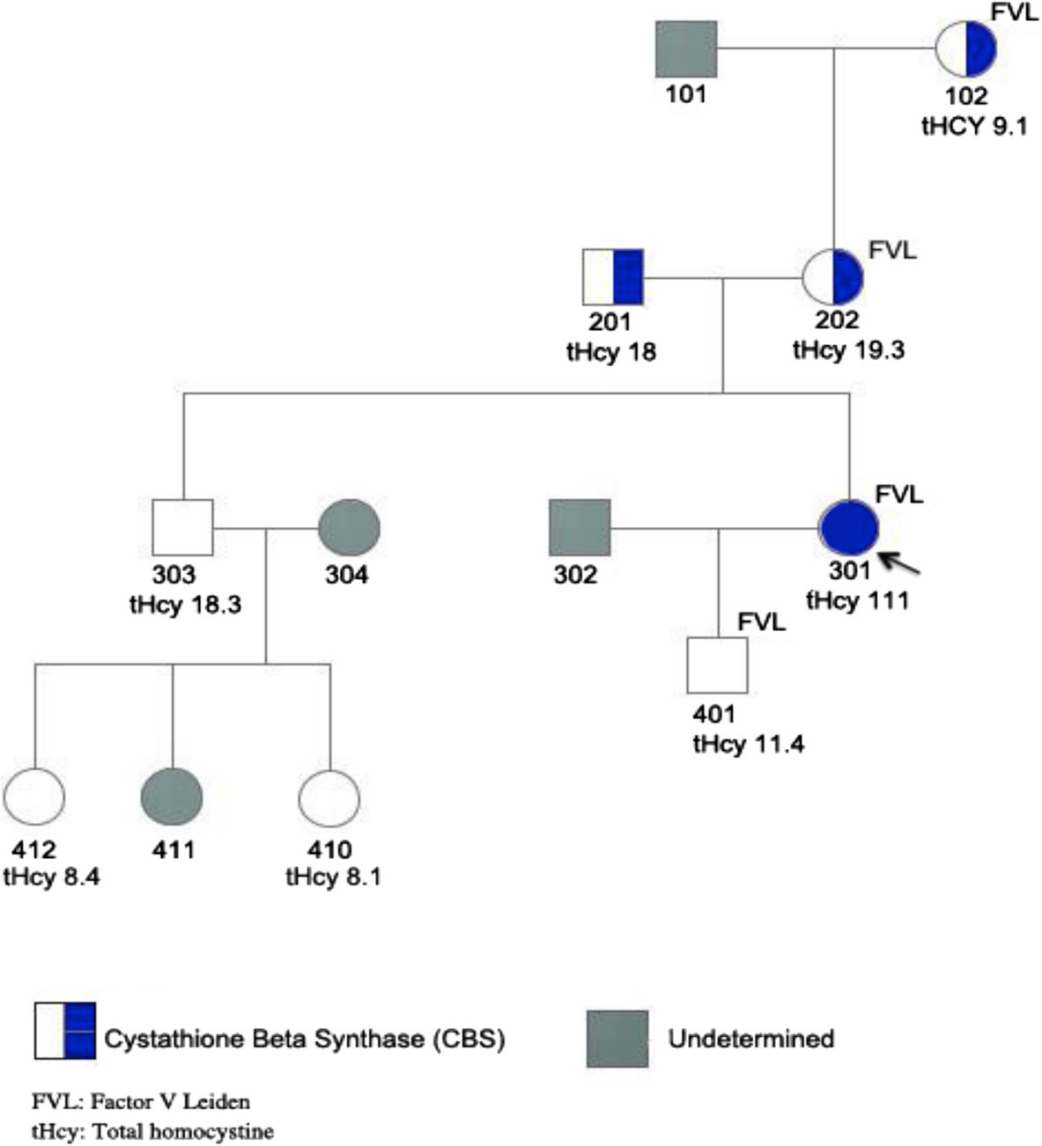
**Kindred showing index patient (301) and her family.** Note homozygosity for the cystathionine beta-synthase gene I278T and co-existant Factor V Leiden.

ID: number refers to Figure 2, LDL: low density lipoprotien cholesterol, tHcy: total homocysteine, CBS: cystathionine β-synthase gene mutation (p.I278T), MTHFR: methylenetetra-hydrofolate reductase C766T single nucleotide polymorphism, FVL: Factor V Leiden mutation.

The remainder of the investigations was within the reference range. Protein C and S, Lipoprotein (a), anticardiolipin antibodies and antithrombin III levels were within normal. Prothrombin gene mutation (20210) was negative.

Her father had a history of a myocardial infarction at the age 50. Neither the patient nor her family members have a Marfanoid habitus, nor joint hyperlaxity or other skeletal anomalies. They do not have lens dislocations or mental retardation. The tHcy level was moderately elevated in both parents. Her mother was heterozygous for FVL mutation.

We performed a skin biopsy and cultured skin fibroblasts to examine known inborn error of cobalamin metabolism. The incorporation of both [^14^C]-propionate and [^14^C]-methyltetrahydrofolate and the uptake of [^57^Co]-cyanocobalamin were normal. The proband had adequate synthesis of both adenosylcobalamin and methylcobalamin. We used a targeted gene sequencing approach to identify the molecular defect. The *MTHFR* gene (OMIM 236250) was sequenced and the common 677C > T polymorphism was found in 4 of 9 family members, although not in the proband (Figure 1). We subsequently sequenced the cystathionine β-synthase gene (OMIM 236200). The proband had a homozygous (p.I278T) mutation in the *CBS* gene, a known vitamin B_6_-responsive mutation [2–4].

In addition to this deleterious mutation at the CBS gene, the patient had coagulation FVL, a combination of findings traits shared by no other family members (Figure 2).

The management of the patient includes folic acid, vitamin B_12_ and vitamin B_6_ as described above, warfarin, targeting an international normalized ratio of 2.0-2.5, a beta-adrenergic blocker for hypertension, and a statin for elevated cholesterol. The patient has remained asymptomatic for nearly twelve years.

## Discussion

Homocysteine promotes vascular disease in part by causing endothelial cell dysfunction and reduced thrombolysis [5,6]. In a meta-analysis of clinical trials, folate supplementation was associated with 25% reduction in homocysteine level but has no effect on vascular outcomes or all-cause mortality. Resistance to activated protein C, typically due to the FVL mutation, is the most common inherited cause of sporadic thrombosis [7]. Thromboembolism occurs in only about one third of patients with homocystinuria, which suggests that other contributory factors are needed for the development of thrombosis. Screening for FVL may be indicated in patients with homocystinuria and their family members [8]. In a study on 24 patients with hyperhomocysteinemia due to CBS deficiency, six patients had a thrombotic event, only one was a carrier for FVL and three were carrier of *MTHFR* 677C > T mutation. These and additional data from Ireland, indicate that FVL is not an absolute determinant of venous thrombosis in homocystinuria caused by CBS mutations [9].

In a large cohort analysis of 19,678 patients with venous thrombosis who underwent thrombophilia screening in Italy, 38 had severe hyperhomocysteinemia (0.2%). In this subgroup the median age at diagnosis was 47 years (range 19-83), and the median level of tHcy was 130 μmol/L (range 101-262). Recurrent thrombosis occurred in 42% of cases [10]. In seven families in a consanguinous Israeli Arab population, 4/45 members were both homozygous for CBS and heterozygous for the FVL mutation, and all four developed deep vein thrombosis. The increased tendency to thrombosis could result from an additive adverse effect of these two defects on a common protective mechanism in the coagulation cascade. The authors concluded that major thrombotic events occurred only in concurrent homocystinuria and FVL mutations, as is seen in our patient [11]. In a Turkish study, one out of six subjects with CBS mutation had.

FVL and developed severe thrombosis leading to amputation of the leg [12]. A study from Italy showed a mutation of CBS gene associated with FVL mutation causes severe deep vein thrombosis despite only mild elevation of the homocysteine level [9]. Consistent with this study, our patient had moderate to severe homocysteinemia and suffered multiple thrombotic events. There is an association between serum homocysteine and preeclampsia consistent with the increase risk of vascular damage and thrombosis. One study that included fifty pregnant women with preeclampsia, found that 54% have hyperhomocysteinemia caused by low vitamin B_12_ and folate [13].

Our patient has recurrent thromboembolic episodes. In line with current guideline, lifelong anticoagulant is warranted. However, since the severe hyperhomocysteinemia is corrected the question whether anticoagulant can be stopped needs to be discussed with the patient.

Homocysteine is also associated with arterial occlusive disease, but this association has been in great part explained by co-variables, such as renal function, male gender and increasing age. In a multi-variate analysis, homocysteine does not appear to be a strong cardiovascular risk factor [14–17]. Clinical trials aimed at preventing cardiovascular diseases have failed to show a reduction in cardiovascular events, and the interest for measuring homocysteine has fallen dramatically. It is not surprising, therefore, that sporadic cases such as the one presented here can have such a dramatic presentation, untreated for many years [11,16,18]. An index of suspicion therefore should alert physicians to measure plasma tHcy levels in unexplained venous thrombo-embolic events. The response to treatment in this case had likely contributed to the patient’s satisfactory clinical evolution over the past twelve years.

## Conclusion

When tHcy levels are consistently very high, evaluation of family members is important, starting with siblings and parents. Some authorities strongly argue that a molecular diagnosis is required as this knowledge may change the therapeutic approach.

## Consent

A written informed consent was obtained from the patient and his family members for the publication of this report and any accompanying images.
